# Improving medication management in multimorbidity: development of the MultimorbiditY COllaborative Medication Review And DEcision Making (MY COMRADE) intervention using the Behaviour Change Wheel

**DOI:** 10.1186/s13012-015-0322-1

**Published:** 2015-09-24

**Authors:** Carol Sinnott, Stewart W. Mercer, Rupert A. Payne, Martin Duerden, Colin P. Bradley, Molly Byrne

**Affiliations:** Department of General Practice, University College Cork, Cork, Ireland; General Practice and Primary Care, Institute of Health and Wellbeing, University of Glasgow, Glasgow, UK; Cambridge Centre for Health Services Research, Institute of Public Health, University of Cambridge, Cambridge, UK; Centre for Health Economics and Medicines Evaluation, Bangor University, Bangor, UK; Health Behaviour Change Research Group, School of Psychology, National University of Ireland, Galway, Ireland

## Abstract

**Background:**

Multimorbidity, the presence of two or more chronic conditions, affects over 60 % of patients in primary care. Due to its association with polypharmacy, the development of interventions to optimise medication management in patients with multimorbidity is a priority. The Behaviour Change Wheel is a new approach for applying behavioural theory to intervention development. Here, we describe how we have used results from a review of previous research, original research of our own and the Behaviour Change Wheel to develop an intervention to improve medication management in multimorbidity by general practitioners (GPs), within the overarching UK Medical Research Council guidance on complex interventions.

**Methods:**

Following the steps of the Behaviour Change Wheel, we sought behaviours associated with medication management in multimorbidity by conducting a systematic review and qualitative study with GPs. From the modifiable GP behaviours identified, we selected one and conducted a focused behavioural analysis to explain why GPs were or were not engaging in this behaviour. We used the behavioural analysis to determine the intervention functions, behavioural change techniques and implementation plan most likely to effect behavioural change.

**Results:**

We identified numerous modifiable GP behaviours in the systematic review and qualitative study, from which active medication review (rather than passive maintaining the status quo) was chosen as the target behaviour. Behavioural analysis revealed GPs’ capabilities, opportunities and motivations relating to active medication review. We combined the three intervention functions deemed most likely to effect behavioural change (enablement, environmental restructuring and incentivisation) to form the MultimorbiditY COllaborative Medication Review And DEcision Making (MY COMRADE) intervention. MY COMRADE primarily involves the technique of social support: two GPs review the medications prescribed to a complex multimorbid patient together. Four other behavioural change techniques are incorporated: restructuring the social environment, prompts/cues, action planning and self-incentives.

**Conclusions:**

This study is the first to use the Behaviour Change Wheel to develop an intervention targeting multimorbidity and confirms the usability and usefulness of the approach in a complex area of clinical care. The systematic development of the MY COMRADE intervention will facilitate a thorough evaluation of its effectiveness in the next phase of this work.

**Electronic supplementary material:**

The online version of this article (doi:10.1186/s13012-015-0322-1) contains supplementary material, which is available to authorized users.

## Background

Multimorbidity, the presence of two or more chronic conditions, affects over 60 % of patients in primary care [1]. In a healthcare system that has evolved around the management of single chronic diseases, this presents major challenges to healthcare provision, research and medical education [2]. In 2014, the US Department of Health and Human Services recognised these challenges by stating the need to better equip clinicians in the management of multimorbidity, making specific reference to medication management [3]. Multimorbidity frequently leads to the prescription of multiple long-term medications [4]. The resulting polypharmacy is an independent risk factor for negative health outcomes such as adverse effects and drug interactions [5]. For prescribers, this creates a tension between keeping the number of medicines to a minimum while still prescribing what evidence-based guidelines advocate as being in the patient’s best interest [6]. This is especially the case for general practitioners (GPs), who must coordinate and oversee the medications prescribed by numerous doctors involved in the care of a multimorbid patient [7].

Despite the prevalence of multimorbidity, few interventions have been developed to improve medication management in this field to date. A recent systematic review, which focussed on interventions to optimise outcomes in patients with multimorbidity in primary care, found only two that specifically addressed medication management. However, both interventions related to enhanced involvement of pharmacists, rather than the prescribing actions of GPs [8]. Thus, the development of interventions to improve GPs’ contribution to medication management in patients with multimorbidity is a priority.

In the past, interventions that aimed to change healthcare professionals’ behaviour have resulted in suboptimal effects, due to a lack of theoretical consideration at the development stage [9]. The UK Medical Research Council (MRC) guidance for the development of complex interventions in healthcare emphasises the importance of using theory in intervention design [10]. However, the MRC document does not put forth any specific suggestions on how to do this which leaves intervention designers, many of whom are interested in theory only to the extent that it can help them achieve improvements in clinical care, with an array of dilemmas [11]. The large pool of available theoretical models means that critical theories may be missed, and there is little clarity on how to choose the most appropriate theory for the behaviour in question [12]. In addition, intervention developers have traditionally had little to guide them on the specification of intervention content [13].

**Fig. 1:**
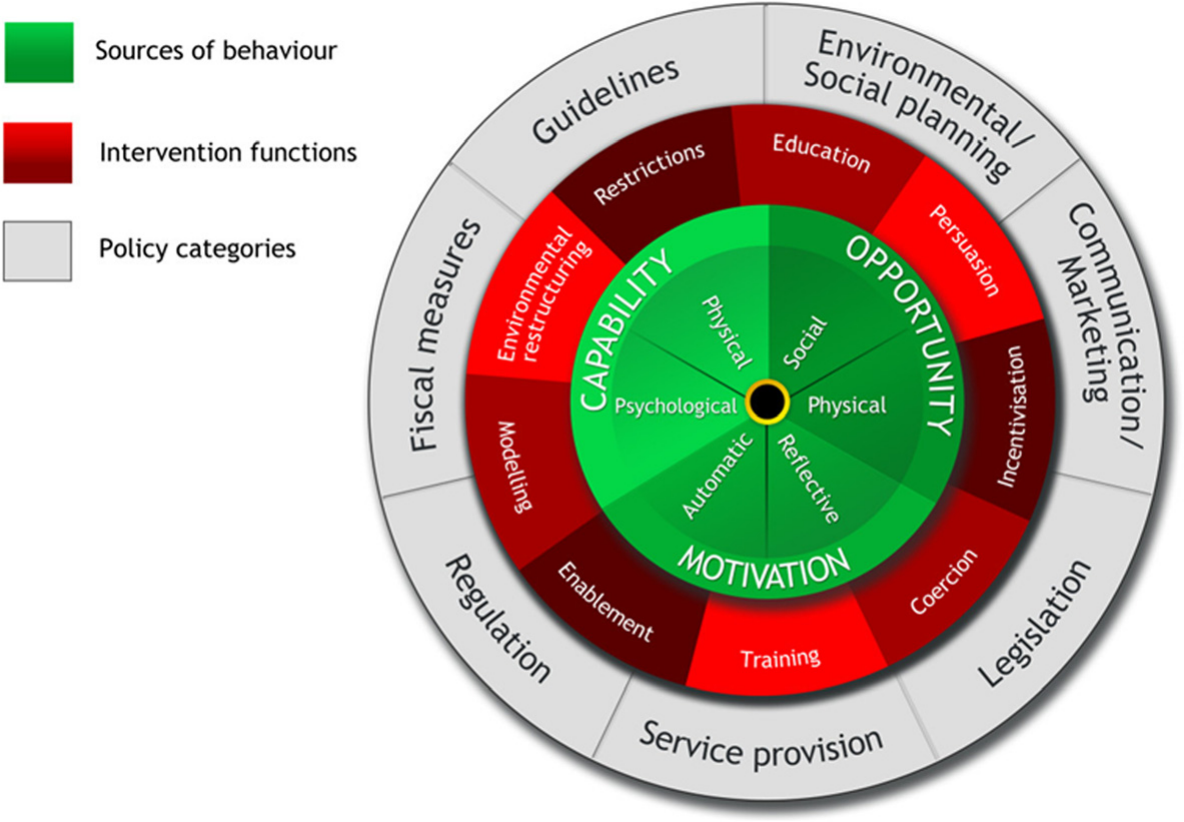
The Behaviour Change Wheel

Over the last few years, this gap has been addressed by an approach known as the Behaviour Change Wheel (BCW), which explicitly integrates behavioural theory with the development and description of behavioural change interventions [14]. A core feature of the BCW is a theoretical model which is used to conduct an analysis of the behaviour in question. The model is based on the hypothesis that the interaction between one’s capability (C), opportunity (O) and motivation (M) can provide explanations for why a particular behaviour (B) is or is not performed (COM-B). Each of these components can be further subdivided (Fig. 1). Capability may be physical (the physical skill, strength and stamina) or psychological (the knowledge or psychological skills, strength or stamina to engage in the necessary mental processes). Opportunity may be physical (afforded by the environment, including resources, locations, time etc.) or social (afforded by interpersonal influences, social cues, and cultural norms that influence the way we think about things). Motivation may be reflective (plans, self-conscious intentions or evaluations) or automatic (reflex responses, impulses, drive states). The COM-B behavioural analysis guides the choice of intervention functions (or strategies) most likely to achieve behavioural change. Additionally, the intervention functions have been linked to a taxonomy of 93 replicable behavioural change techniques [15], and those techniques particularly suitable for each intervention function have been highlighted [14]. Following this structured approach lends transparency to the process of intervention development and facilitates its subsequent implementation and evaluation [12].

Since its original publication in 2011, the BCW has received a lot of academic interest, and a number of groups have already used it to develop or study the implementation of interventions by healthcare professionals [16–19]. To our knowledge, there are no published examples of using the BCW to develop a de novo intervention targeted at healthcare professionals in the complex field of multimorbidity. As the application of the BCW may vary according to the setting and target behaviour, examples of the generalizability of the approach are required. Furthermore, published examples of its use will contribute to the ongoing development and refinement of the method.

In this paper, we describe the development of an intervention to improve medication management in multimorbidity by GPs, in which we applied the steps of the BCW to enable a more transparent implementation of the MRC framework for design and evaluation of complex interventions.

## Methods

In the MRC framework, intervention development comprises three stages: identifying the evidence base, identifying and applying appropriate theory to the available (and if necessary, new) evidence and modelling process and outcomes [10]. Like the MRC framework, the BCW [14] also has three broad stages but they involve different tasks (i.e. understanding the behaviour, identifying intervention options and identifying content and implementation options) and are subdivided into a further eight steps (i.e. defining the problem in behavioural terms, selecting the target behaviour, specifying the target behaviour, identifying what needs to change, identify appropriate intervention functions, identifying policy categories, identifying behavioural change techniques and determining the mode of delivery) [14]. As we were using the BCW within the overarching framework of the MRC, we mapped the eight BCW steps directly on to the three development stages of the MRC to enhance the clarity and generalizability of our approach (see Table 1).

**Table 1:** Mapping steps of Behaviour Change Wheel to the three stages of intervention development in the UK Medical Research Council guide on complex interventions in healthcare

MRC development stage [10]	BCW steps [14]	BCW stages
1. Identify the evidence base	1. Define the problem in behavioural terms	1. Understand the behaviour
	2. Select the target behaviour	
	3. Specify the target behaviour	
2. Identify/develop theory	4. Identify what needs to change	
	5. Identify appropriate intervention functions	2. Identify intervention options
	6. Identifying policy categories	
3. Model process and outcomes	7. Identifying behavioural change techniques	3. Identify content and implementation options
	8. Determine the mode of delivery	

### MRC stage 1: identifying the evidence base

To begin, we reviewed the existing evidence on medication management in multimorbidity and supplemented this with new evidence in order to clearly define our problem of interest and then select and specify the behavioural target for intervention.

#### BCW step 1: define the problem in behavioural terms

We searched for relevant published literature, in particular existing systematic reviews, to help us understand the problems associated with medication management in multimorbidity in primary care. While we identified two relevant quantitative reviews [8, 20], we also found a number of pertinent qualitative studies. Therefore, we conducted a systematic review and synthesis of the relevant qualitative evidence, the methods of which have been published elsewhere [21].

We addressed the gaps identified from the qualitative synthesis by conducting a qualitative interview study with GPs, specifically to generate further information on their approaches to prescribing in multimorbidity. The methods for the interview study have also been published elsewhere [22].

#### BCW step 2: select the target behaviour

From the aggregated qualitative synthesis and interview data, we (CS and CB) identified the modifiable GP behaviours relating to medication management in multimorbidity and selected one key behaviour to target in our intervention. This judgement was informed by criteria set out in the BCW guide which are the likelihood that behavioural change would be implemented, the likely impact of changing the behaviour, the spillover or knock on effect of change on other behaviours and the ease with which each behaviour could be measured [14].

#### BCW step 3: specify the target behaviour

Once the target behaviour was decided, we specified in greater detail what and who needs to change and where and when this change should happen.

### MRC stage 2: identifying/developing theory

In the next stage, we used the COM-B (capability, opportunity, motivation—behaviour) model to develop a theoretical understanding of the target behaviour and guide our choice of intervention functions.

#### BCW step 4: identify what needs to change to achieve the desired behaviour

We used the COM-B model to frame our qualitative behavioural analysis of the qualitative synthesis and interview data. We (CS and CB) coded empirical data relevant to GPs’ psychological and physical capabilities (C), social and physical opportunities (O) and reflective and automatic motivations (M) to highlight why GPs were or were not engaging in the target behaviour and what needed to change for the target behaviour to be achieved. Where multiple COM-B components were potentially relevant to one section of the data, the component whose definition (as set out in the BCW guide, [14]) best fit the context of our data was chosen. The results of this analysis was presented to the other authors at a consensus meeting and refined accordingly.

#### BCW step 5: identify intervention functions to achieve the desired behaviour

The BCW incorporates a comprehensive panel of nine intervention functions, shown in Fig. 1, which were drawn from a synthesis of 19 frameworks of behavioural-intervention strategies. We determined which intervention functions would be most likely to effect behavioural change in our intervention by mapping the individual components of the COM-B behavioural analysis onto the published BCW linkage matrices [14]. Each intervention function seen to be potentially relevant to our data was considered in detail. We used the affordability, practicability, effectiveness and cost-effectiveness, acceptability, side effects/safety and equity (APEASE) criteria, another component of the BCW approach, to grade the potentially relevant intervention functions into first and second line options [14].

#### BCW step 6: policy categories

The BCW also includes matrices which signpost the seven broad policy-level interventions for achieving behavioural change, shown in Fig. 1. As we were not primarily concerned with changing policy in this study, we did not undertake this step in detail, other than listing the options that may be relevant to levering our intervention in the future.

### MRC stage 3: modelling process and outcomes

In this third stage, we specified our intervention content in more detail and identified an appropriate way of implementing the intervention within our context.

#### BCW step 7: identify behavioural change techniques

The selected intervention functions represented our broad approach to achieving behavioural change, but we required fine-grained techniques to operationalise these functions. We used the links previously drawn between the BCW and the taxonomy of 93 behavioural change techniques [14, 23] to list those techniques most frequently used with our selected intervention functions. We held an expert panel consensus meeting to review the suitability of each of these techniques, in the light of our previously collected qualitative data, the context of the intervention and by referring to the APEASE criteria. Each member of the panel had expertise in one or more areas of relevance (clinical pharmacology and prescribing (CB, MD, RP), general practice (CB, CS, MD, RP, SM), behavioural science and intervention design (MB) and multimorbidity (CS, RP, SM)).

#### BCW step 8: identify mode of delivery

As we were developing an intervention to be implemented by individual GPs, this step (mode of delivery) required explicit consideration of implementation in the heterogeneous setting of general practice. We used the expert panel consensus to specifically address modelling questions posed in the MRC framework which were would it be possible to use this, what subgroup of patients should it be used for, what outcomes should be sought and what are the facilitators/obstacles at practice level [10]. If multiple implementation options existed, agreement was reached by discussing each option, with reference to the APEASE criteria [14].

## Results

### MRC stage 1: identifying the evidence base

#### BCW step 1: define the problem in behavioural terms

We identified two existing systematic reviews which were relevant. Patterson et al. reviewed existing interventions to improve prescribing and polypharmacy in older patients [20]. Only one of the included studies involved GPs and showed that computer decision support reduced inappropriate drug initiation in primary care [24]. The authors suggested that future polypharmacy interventions must address the complexity of clinical situations and the individuality of prescribers. Smith et al. reviewed interventions to improve patient outcomes in multimorbidity in primary care. Two included studies addressed medication management but these involved pharmacists rather than GPs. Here, the authors suggested that future interventions should target specific problems relating to multimorbidity, be integrated into existing healthcare systems and be embedded with inter-professional collaboration [8].

Our qualitative synthesis included ten studies from seven countries involving a total of 275 GPs [21]. A key theme was GPs’ sense of professional isolation in the management of multimorbid patients. This emanated from the interplay between four aspects of the management of patients with multimorbidity: (i) the disorganisation and fragmentation of healthcare between primary and secondary care, (ii) the inadequacy of guidelines and evidence-based medicine for multimorbidity, (iii) challenges in delivering patient-centred, rather than disease-focused, care and (iv) barriers to shared decision-making.

In the qualitative interview study, we interviewed 20 GPs about 51 multimorbid cases [22]. We found that GPs responded to clinical dilemmas in multimorbidity by ‘satisficing’, i.e. accepting care that they deemed satisfactory and sufficient for a particular patient, yet acknowledging that aspects of that care may not be optimal. In patients with changing disease trajectories, satisficing was manifested as relaxing targets for disease control, negotiating compromise with the patient, or making ‘best guesses’ about the most appropriate course of action to take. In multimorbid patients perceived as stable, GPs’ default approach was to ‘maintain the status quo’ rather than actively rationalise medications.

#### BCW step 2: select the target behaviour

**Fig. 2:**
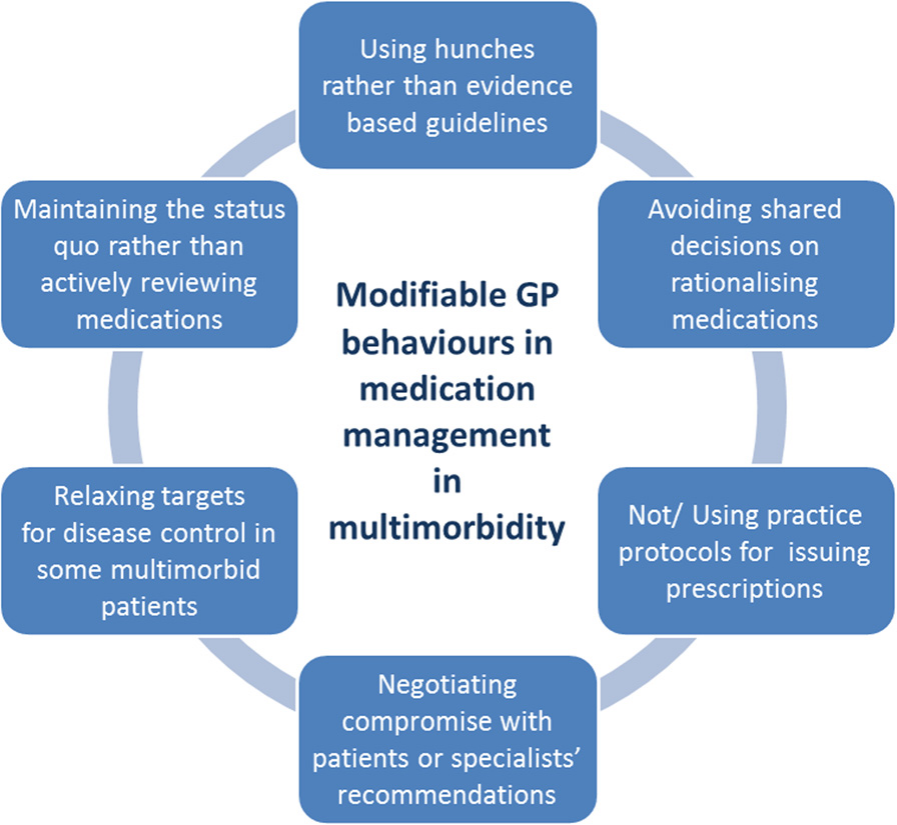
Modifiable GP behaviours in medication management in multimorbidity identified in qualitative synthesis [21] and interview study [22]

The numerous modifiable GP behaviours relating to medication management in multimorbidity are shown in Fig. 2. ‘Maintaining the status quo’ was observed in all of the qualitative interviews despite best practice guidelines which state that patients receiving long-term medicines need medication reviews at regular intervals. Targeting this behaviour would likely result in behavioural change as the qualitative study showed GPs extant discomfort with it [22]. Furthermore, it would be desirable to see GPs adopt a less-passive approach to medication management even if it did not always lead to downstream changes to medications. There was a high possibility of ‘spill over’ of the actions of medication review for multimorbidity into other prescribing activities. Lastly, changing this behaviour would be relatively easy to measure. We judged that the other modifiable behaviours were not as attractive: adopting practice protocols would have a big impact and high spillover, but given current financial and staffing pressure on practices would be a difficult organisational change to achieve; relaxing targets and negotiating compromise may be argued to be appropriately patient-centred in multimorbidity and trying to change these, in a healthcare system where progressively less appears to be patient-centred, may be resisted by GPs; addressing shared decision-making has merit but requires interventions targeting GPs’ communication skills (rather than prescribing) which was not our specific focus.

#### BCW step 3: specify the target behaviour

The target behaviour was specified as active, purposeful medication review instead of passive ‘maintaining the status quo’ for patients with multimorbidity, to be conducted by GPs, in routine general practice, on a regular basis.

### MRC stage 2: identifying/developing theory

#### BCW step 4: identify what needs to change to achieve the desired behaviour

We used COM-B to identify GPs’ capabilities (C), opportunities (O) and motivations (M) for engaging, or not engaging, in active medication review. The themes that emerged from this analysis are shown in Table 2, with illustrative quotes from the qualitative synthesis and the interview study. For example, GPs adopted a passive approach to medication management due to their uncertainty (lack of psychological capability) about which medications were most valuable in patients with multimorbidity, especially given the absence of satisfactory guidelines in this field. Insufficient time within the consultation led to a lack of physical opportunity to review medications. GPs also found medication review difficult because of a cultural milieu which holds that treatment for chronic disease is lifelong (lack of social opportunity). This was especially the case if the patient had been compliant with their medications for many years. Many GPs had developed a habitual response to ‘not rock the boat’ in patients with multimorbidity, an approach which involved not making changes to medications unless there was a pressing reason to do so. This response was reinforced by their experiences of the negative consequences of stopping or changing medications for patients with multimorbidity in the past (automatic motivation). GPs’ reflective motivations against medication review included the opportunity cost of using their professional time for this purpose and a fear of negative consequences from rationalising medications. GPs also had motivations to review medications which included improving patient outcomes, reassuring themselves that they are delivering best care, and guarding against medico-legal repercussions.

**Table 2:** Behavioural analysis, selected intervention functions and behavioural change techniques, referencing empirical data from the qualitative synthesis (QS) and the interview study (IS)

	ᅟ

#### BCW step 5: identify intervention functions

We found that all nine intervention functions listed in the BCW were relevant to our behavioural analysis. Additional file 1 shows our assessment and grading of each intervention function using the APEASE criteria into first and second line options. The three intervention functions *most* relevant for our intervention were enablement, environmental re-structuring and incentivisation. The relationship between the components of the COM-B behavioural analysis and the three selected intervention functions are shown in Table 2.

#### BCW step 6: policy categories

The broad policy options, signposted by the BCW matrices as being potentially useful for achieving behavioural change, were communication/marketing, service provision policy, legislation, guidelines and regulation.

### MRC stage 3: modelling process and outcomes

#### BCW step 7: identify behavioural change techniques

From the taxonomy of 93 behavioural change techniques, we listed the techniques most frequently used to deliver the three intervention functions we had selected [14, 23]. The resulting 32 potentially relevant techniques are listed in Additional file 2. In the expert panel, we reviewed how each technique could be applied to the context of medication management in multimorbidity. The panel’s choice of techniques was influenced principally by the key findings of the qualitative studies: GPs’ sense of isolation in the management of multimorbid patients revealed in the qualitative synthesis [21], and GPs’ lack of certainty and efforts to ‘share the onus of responsibility’ seen in the interview study [22]. Thus, we focused on options that would enhance GPs’ means of professional support. Although enhanced communication between GPs and pharmacists is being investigated in other healthcare systems [25, 26], it is not currently an option in Irish general practice due to the lack of community pharmacists. Similarly, communication between GPs and the specialists involved in multimorbidity patient care was seen in both qualitative studies to be fraught with poor access and a single-disease approach. A useful source of support for some GPs in the interview study was their GP colleagues. These interactions occurred on an informal basis within practices and were notable for their ready accessibility and generalist nature [22]. We were unaware of any work exploring collaborative decision-making between GPs in multimorbidity, so focused on this approach. From the list of 32, we considered which techniques would pragmatically facilitate collaborative decision-making between GPs. Additional file 2 shows how many were quickly eliminated as not being relevant to the context or purpose of the intervention. The five techniques eventually selected as ‘active ingredients’ were social support (practical), restructuring the social environment, use of prompts/cues, action planning and self-incentives. The definition of each technique and qualitative data to support their selection are shown in Table 2. The combination and integration of each technique into the overall intervention, named MultimorbiditY COllaborative Medication Review And DEcision Making (MY COMRADE), is shown in Table 3.

**Table 3:** Description of final intervention

The final intervention is called MultimorbiditY COllaborative Medication Review And DEcision Making (MY COMRADE)
It involves the following (relevant behavioural change techniques are in brackets):
GPs will be asked to schedule protected time for themselves and one of their GP colleagues to conduct the collaborative medication review and enter this time into the practice appointment book. They will be asked to choose a day/time/office that suits them best and decide how many patient cases to review in one sitting (action planning). The GPs will choose multimorbid patients from their caseload and in the scheduled review time will review medications, supported by their GP colleague (social support and restructuring social environment). The medication review will be cued by the seven prompts described in the NO TEARS [27] medication checklist (prompts and cues). GPs will be asked to record recommendations for medication change that arise from the review in the patient’s notes to allow them to discuss these with the patient during their next consultation. After completing the review, GPs will award themselves continuing professional development points: one point for each cumulative hour of the activity completed (self-incentives).

#### BCW step 8: identify mode of delivery

In the expert panel meeting, we then formulated an intervention implementation plan. Four specific aspects of implementation were reviewed, and the various options considered for each aspect are fully described in Additional file 3. In summary, the following implementation plan was formulated:

1. What prompts should be used to guide medication review in MY COMRADE?
  After reviewing eight different prescribing tools and checklists (listed in Additional file 3), it was agreed that a modified version of the seven prompts in the NOTEARS [27] checklist for medication review would be used to cue the review.
2. How should GPs choose which patients to review using MY COMRADE?
  After reviewing multiple options (see Additional file 3), it was agreed that GPs should choose patients prescribed ten or more regular medicines or four or more medicines with at least one other complicating factor (i.e. meets criteria for potentially inappropriate prescribing, at risk of a well-recognised drug-drug interaction, has poor adherence or receiving end-of-life or palliative care), in line with recommendations from the Kings Fund report on Polypharmacy and Medication Optimisation [28].
3. How should the behavioural change technique “action planning” be operationalised?
  One of the behavioural change techniques, action planning, specifically relates to implementation and was selected to account for the wide variety of structures and systems that occur in general practice. Each GP will be given clear guidance on how to tailor MY COMRADE to suit their practice. This will involve asking them to choose a particular day, time of the day and office in which to do the review. They will decide on the number of cases to review in one sitting, and the GP pairs that will conduct reviews within a practice. In advance of trialling MY COMRADE, GPs will be asked to consider what they envision as problematic for its implementation, and how these problems could be tackled, knowing their own practice.
4. How should the intervention be evaluated?
  The initial evaluation will focus on intervention implementation (i.e. did medication review take place?). The behavioural change techniques and other causal or contextual mechanisms associated with behavioural change will be determined using qualitative methods. If MY COMRADE is shown to be effective, future evaluations will assess other health outcomes such as the number of/type of medication changes made and changes in rates of healthcare utilisation.

## Discussion

This paper describes the systematic, structured development of an intervention to improve medication management for multimorbid patients by GPs. The intervention is called MY COMRADE. It is, to our knowledge, the first intervention directed at the management of multimorbidity in primary care, developed by using the Behaviour Change Wheel to clearly implement the framework of the MRC guide on complex interventions.

MY COMRADE involves collaborative decision-making by two GPs who support each other in the review of medications prescribed to a complex multimorbid patient, guided by cues which relate to safe prescribing. The broad functions of the intervention (enablement, environmental restructuring and incentivisation) are theoretically based. These functions will be achieved using five specific behavioural change techniques: social support (practical), restructuring the social environment, use of prompts/cues, action planning and self-incentives. The technique of collegial social support is a crucial feature of our intervention, which we expect will greatly enable GPs’ capabilities in conducting active medication reviews. It may be particularly important in de-prescribing medications or prioritising patient-centred rather than disease-focused care in multimorbidity which are challenging aspects of medication management, not least because of the fear of litigation which this intervention may now help ameliorate.

### Comparison with other work

Since its publication in 2011, the BCW has been used in the development of interventions targeting healthcare professionals in a variety of ways. For example, Alexander et al. used COM-B to understand barriers and enablers to preventative health examinations for young children in Australian general practice, with a view to designing an implementation intervention to increase the conduct of these examinations [16]. They did not describe later steps of the BCW, such as choice of intervention functions, and did not describe in detail how their implementation intervention would look. In contrast, we used the BCW to highlight areas for improvement in professional practice and then develop an intervention targeted to these areas, rather than simply increasing the implementation of a pre-existing intervention.

Murphy et al. used COM-B to develop a capacity-building programme to enhance pharmacists’ roles in mental health care [19]. This group felt that implementation processes must be prioritised during the early stages of intervention development, and they wove theories of behavioural change and implementation together in an iterative way. While we agree that implementation should be considered at all stages of development work, we did not find it necessary to use a specific implementation framework. The initial steps of the BCW revealed multiple areas for improvement in GPs’ professional practice. Once one had been chosen, the remaining steps of the BCW involved developing an implementation intervention to enhance the performance of this desired behaviour. Additionally, by incorporating the behaviour change technique of action planning, implementation was explicitly integrated into our intervention. Action planning requires an individual GP to plan the frequency, duration and intensity of the planned intervention activity [14]. Thus, rather than a prescriptive implementation strategy, action planning will allow each GP to adapt the intervention for use within their own practice. The variation in implementation, as well as fidelity to other behavioural change techniques, will be evaluated in the next phase of this work and will help to inform the debate on optimal approaches to implementation planning in intervention development.

### Strengths and weaknesses

We began this work with the broad aim of developing an intervention to improve medication management in multimorbidty, but we did not have a predefined idea of what the intervention would be at the outset. Adhering to the guidance of the MRC by using a theoretical approach, which was chosen a priori, lent direction, structure and transparency to this process in multiple ways.

First, the MRC states the need to identify the evidence base and supplement this with new evidence if necessary. In doing this, we generated much needed data on the management of medications in multimorbidity, increased our understanding of the problematic areas experienced by GPs and revealed how they currently respond to these difficulties. Second, we then used this empirical data to directly influence the development of the intervention. Following the steps of the BCW allowed us to develop a list of options for behavioural change and to clarify what we were, and what we were not, trying to achieve. Third, we benefitted from using the links between the BCW model and the taxonomy of behavioural change techniques. The taxonomy highlighted novel strategies for behavioural change, many of which we would heretofore not have considered. Although only five techniques are ultimately included in the description of the final intervention, many of the others influenced other aspects of intervention development and the implementation strategy.

Despite the highly systematic and structured approach of the BCW, there are challenges associated with its use and it is not a magic bullet for intervention development. For example, the researcher must make a series of subjective and pragmatic decisions throughout the process. These ‘real life’ decisions can seem at odds with the scientific approach. To counter this and to improve the transparency and generalizability of our methods, we recorded in detail the multiple options available to us at each step of the BCW and expanded on why options were or were not taken.

Furthermore, the multiple steps of intervention development involved a lengthy process: from the beginning of our systematic review to the final refinements of the intervention spanned almost 3 years. Such a prolonged course must be factored in by those pursuing and funding evidence-based intervention development. Other intervention developers have used a ‘top-down’ approach of applying classical behavioural theories such as social cognitive [29] or control theory [30] to inform their choice of intervention functions and behavioural change techniques. In contrast, we employed a ‘bottom-up’ approach to theory development in which the framework of the BCW guided our use of existing evidence and our own qualitative explorations. This led to an intervention which was logical and practical yet still theoretically based.

In addition to the COM-B, the BCW also includes an optional, more detailed framework for behavioural analysis known as the Theoretical Domains Framework [14]. After completing our intervention development, we conducted a validation using the Theoretical Domains Framework (see Additional file 4) which reassuringly demonstrated similar associations between our qualitative data, and our chosen intervention functions and behavioural change techniques.

### Implications for future research

We used the BCW as a lens for viewing GP behaviour, understanding what needed to shift and determining how this shift could be achieved. Our experience confirms the usefulness and generalizability of this approach. Multimorbidity presents many challenges to GPs, particularly relating to the conflicts between patient-centred and disease-focused care but the BCW approach was not hampered by these complexities. Based on our experience, the method is potentially applicable to intervention developers across disciplines as long as sufficient contextual and empirical data exists or can be generated.

Throughout this study, we adhered to the ‘less is more’ maxim of intervention design [14]. We could have taken a more complex multi-faceted approach, such as incorporating other stakeholders, i.e. pharmacists or specialists. Instead, we adopted the recommendations from the systematic review by Smith et al. that changes targeting specific problems are more likely to be effective [8]. Smaller changes can be achieved, sustained and built upon in future interventions, and substantial behavioural change is more likely to result from the aggregation of these smaller changes [14]. We applied the same tenets to our assessment of outcomes—rather than initially looking at downstream effects such as changes in prescribing, we will concentrate first on proximal changes such as implementation of the intervention. Once we are assured that it is acceptable, feasible and leads to behavioural change, we can assess outcomes in prescribing safety and polypharmacy at a later stage.

To date, there is limited evidence available on which behavioural change techniques are most effective in specific settings. We expect that characterising the active components in the MY COMRADE intervention using the taxonomy of behavioural change techniques [15] will aid implementation and replication of the intervention. The clear specification of the intervention will also facilitate a thorough evaluation of the impact of the selected behavioural change techniques and will help to inform evidence-based strategies for intervention development in the future.

In this study, we did not undertake the sixth step of the BCW relating to policy options in detail. However, if the intervention is shown to be effective in our ongoing feasibility and pilot work, scaling-up of the intervention will require greater consideration of the external context of healthcare policy and widespread implementation.

## Conclusion

This paper describes the development of an intervention to improve medication management in multimorbidity by GPs. The intervention, which is called MY COMRADE, is based on purposively collected data on behaviour in context and a novel approach to intervention design, the Behaviour Change Wheel. While the Behaviour Change Wheel is not a magic bullet for intervention design, this paper confirms the usability and usefulness of this approach in a complex area of clinical care. The systematic, transparent approach used in the development of the MY COMRADE intervention will facilitate a thorough evaluation of its effectiveness in the next phase of this work.

## Additional files

Additional file 1:
**BCW step 5: identify intervention functions using APEASE criteria.** (DOCX 26 kb) Additional file 2:
**BCW step 7: identify behavioural change techniques.** (DOCX 24 kb) Additional file 3:
**BCW step 8: identify mode of delivery using expert panel.** (DOCX 33 kb) Additional file 4:
**Validation of the chosen intervention functions and behavioural change techniques using the theoretical domains framework.** (DOCX 32 kb)

## Abbreviations

BCW: Behaviour Change Wheel
COM-B: capability, opportunity, motivation—behaviour. Refers to a model comprised of three components—capability, opportunity, motivation—that are necessary for a given behaviour to occur
GP: general practitioner
MRC: Medical Research Council
