# Supplementary material for: Improving medication management in multimorbidity: development of the MultimorbiditY COllaborative Medication Review And DEcision Making (MY COMRADE) intervention using the Behaviour Change Wheel
Source: Implement Sci. 2015 Sep 24;10:132. doi: 10.1186/s13012-015-0322-1 (PMC4582886; doi:10.1186/s13012-015-0322-1)
Supplement: Additional file 1: — BCW step 5: identify intervention functions using APEASE criteria. (DOCX 26 kb) [file 13012_2015_322_MOESM1_ESM.docx]

# Additional file 1

# BCW Step 5: Identify intervention functions using APEASE criteria

Based on the causal analysis of behaviour, all of the nine intervention functions listed in the BCW were signalled as being potentially relevant to our intervention. We used the APEASE criteria [[1](#_ENREF_1)] to judge the merit of each intervention function individually. The acronym stands for affordability, practicability, effectiveness and cost effectiveness, acceptability, side effects/ safety and equity. Using this process we determined our favoured first line and second line approaches. The chosen approaches had a more immediate link between the intervention function and behavioural change.

| BCW Intervention Functions | Affordability | Practicability | Effectiveness &  cost effectiveness | Acceptability | Side effects/ unwanted consequences | Equity | Comments | Decision:  First line  Second Line  Not appropriate |
| --- | --- | --- | --- | --- | --- | --- | --- | --- |
| Incentives | ✓ | ✓ | ✓ | ✓ | 🗶 | ✓ | Creating an expectation of award is a crucial characteristic for the intervention, given the competing demands on GPs time, and the lack of existing recognition for medication reviews. Financial incentives may be effective in changing healthcare professional practice [[2](#_ENREF_2)]. However, care must be taken that the incentive chosen must be affordable and widely available (equitable). As the incentive is for the behaviour of reviewing medications, not the outcome of stopping or reducing medications, unintended consequences on prescribing are unlikely. | First line |
| Environmental Restructuring | ✓ | ✓ | ✓ | ✓ | 🗶 | ✓ | Changing the existing social environment in necessary, to make time/space available to conduct medication review in a safe and systematic way. Adding low cost, generalizable paper-based prompts to the environment is affordable, acceptable and will be effective is appropriate prompts are chosen. | First line |
| Enablement | ✓ | ✓ | ✓ | ✓ | 🗶 | ✓ | Increasing GPs capability by reducing barriers is acceptable, affordable and will be effective if practical, evidence based barriers are addressed. | First line |
| Education | 🗶/- | ✓ | 🗶 | ✓ | ✓ | 🗶 | Increasing GPs’ knowledge through educational programmes is practicable and acceptable: numerous such programmes already exist. However, delivering information on medication review would unlikely be implemented directly without further interventional support; for example, prior educational interventions on prescribing were only effective if consideration was given to local context [[3](#_ENREF_3)]. Also, putting excessive educational focus on the rationalisation of medications may lead to unintended consequences in prescribing. Access to educational programmes may be inequitable/ unaffordable. Educational meetings alone are unlikely to effectively change complex behaviours [[4](#_ENREF_4)]. | Second line |
| Training | 🗶/- | 🗶 | 🗶 | 🗶 | ✓ | 🗶/- | There is a lack of *clinical* prescribing tools for medication review in multimorbidity. Together with the myriad combinations of drugs and diseases that can occur in multimorbidity, this would make it difficult to develop and deliver training programmes. Similar to education, equitable access and affordability cannot be guaranteed. Training in other aspects of medication management in multimorbidty, i.e. communication skills on de-prescribing, may be useful for other interventions but we are focusing on the conduct of medication reviews. | Second line |
| Restriction | 🗶 | 🗶 | 🗶 | 🗶 | ✓ | 🗶/- | This function concerns using rules to increase the target behaviour (medication review) by reducing the opportunity to engage in competing behaviours. Thus, this is not practicable as we are trying to encourage a behaviour that does have a direct competing behaviour. | Not appropriate |
| Coercion | ✓ | 🗶 | 🗶 | 🗶 | ✓ | ✓ | In Ireland, chronic disease care is currently not remunerated under GPs contract of service, so it would not be possible to withhold payments *etc.* for it. Creating an expectation of punishment or limiting access to certain categories of drugs without evidence of medication review would not be acceptable to GPs. | Not appropriate |
| Persuasion | ✓ | 🗶 | 🗶 | 🗶 | 🗶 | ✓ | As most GPs in the qualitative study already agreed with the need for medication reviews, trying to further persuade them of the benefits would be unlikely to stimulate any sustained behavioural change. | Not appropriate |
| Modelling | ✓ | 🗶 | 🗶 | 🗶 | ✓ | ✓ | Using local opinion leaders as an example for GPs to aspire to is inappropriate in this context[[5](#_ENREF_5)], particularly considering the myriad permutations of diseases, and the need for patient-centred decision-making in multimorbidity. | Not appropriate |

1. Michie S, Atkins L, West R: *The Behaviour Change Wheel: A Guide to Desiging Interventions.* Great Britain: Silverback Publishing; 2014.

2. Flodgren G, Eccles MP, Shepperd S, Scott A, Parmelli E, Beyer FR: **An overview of reviews evaluating the effectiveness of financial incentives in changing healthcare professional behaviours and patient outcomes.** *Cochrane Database Syst Rev* 2011**:**CD009255.

3. Arnold SR, Straus SE: **Interventions to improve antibiotic prescribing practices in ambulatory care.** *Cochrane Database Syst Rev* 2005**:**CD003539.

4. Forsetlund L, Bjorndal A, Rashidian A, Jamtvedt G, O'Brien MA, Wolf F, Davis D, Odgaard-Jensen J, Oxman AD: **Continuing education meetings and workshops: effects on professional practice and health care outcomes.** *Cochrane Database Syst Rev* 2009**:**CD003030.

5. Flodgren G, Parmelli E, Doumit G, Gattellari M, O'Brien MA, Grimshaw J, Eccles MP: **Local opinion leaders: effects on professional practice and health care outcomes.** *Cochrane Database Syst Rev* 2011**:**CD000125.
