# Supplementary material for: Improving medication management in multimorbidity: development of the MultimorbiditY COllaborative Medication Review And DEcision Making (MY COMRADE) intervention using the Behaviour Change Wheel
Source: Implement Sci. 2015 Sep 24;10:132. doi: 10.1186/s13012-015-0322-1 (PMC4582886; doi:10.1186/s13012-015-0322-1)
Supplement: Additional file 2: — BCW step 7: identify behavioural change techniques. (DOCX 24 kb) [file 13012_2015_322_MOESM2_ESM.docx]

# Additional file 2

# BCW Step 7: Identify behavioural change techniques

| **Intervention functions chosen as useful for our intervention** | **Potential behavioural change techniques associated with the intervention function** | **Elaboration on how the behavioural change technique could be used to encourage GPs to conduct medication reviews, based on the definition of that behavioural change technique[**[**1**](#_ENREF_1)**].** |
| --- | --- | --- |
| Incentivisation | Self-monitoring of behaviour/outcome of behaviour | Establish a method for the GP to monitor and record their conduct of medication reviews/ outcomes of their medication reviews as part of a behaviour change strategy. In itself and in the current climate in general practice, where medication review is not a priority for GPs, this behavioural change technique is unlikely to motivate GPs without associated initiative or reward. |
|  | Feedback on the behaviour/ outcome(s) of the behaviour | Monitor and provide informative or evaluative feedback on performance of medication review/ outcome of performance of medication review. May be successful, as monitoring of GPs prescribing of benzodiazepines is an already utilised, and accepted, method of addressing benzodiazepine prescribing. Unless there was imminent reward or punishment associated with the behaviour, it may be difficult to initiate. |
|  | Monitoring of behaviour/outcome of behaviour by others with feedback | Observe or record medication review/ outcomes of medication review with the GP's knowledge as part of a behaviour change strategy. In current climate, unlikely to motivate GPs unless there was associated initiative or reward, or a threat of punishment for failing to conduct medication reviews. |
|  | Material incentive | Inform that payment of money, or other valued objects will be delivered if and only if there has been effort and/or progress in performing medication reviews. This would likely be very successful in changing GP behaviour (e.g. QOF initiatives in UK) but is outside the scope of our resources. |
|  | Material reward | Arrange for the payment of money, or other valued objects if and only if there has been effort and/or progress in performing the medication reviews. This would likely be very successful in changing GP behaviour (e.g. QOF initiatives in UK) but is outside the scope of our resources. |
|  | Discrepancy between current behaviour and goal | Draw attention to the discrepancies between a GP’s current behaviour regarding medication review and their previously set action plans, outcome or behavioural goals. Most GPs want to do the best by their patients regarding medications, and many believe in regular medication reviews for the purpose of minimizing treatment burdens as seen in the qualitative study. Given the current pressures on time being experienced by GP, highlighting their shortcomings in the area of medication review is a negative approach. In the short-term, it may dissuade GPs from becoming involved in the feasibility study of the intervention. |
| Enablement | Social support (unspecified/ practical): | Advise or provide practical help for GP for the performance of medication review (e.g. GP colleagues). From our date, many GPs were already engaging in informal conversation with their GP colleagues on how to manage challenging or complex patients, so this avenue is worth exploring as useful |
|  | Reduce negative emotions | Advise GPs on ways of reducing negative emotions (i.e. frustrations/ stress/ uncertainty) to facilitate performance of medication review. Current behaviour (maintaining the status quo) occurs to some extent because GPs are avoiding these negative emotions. Tackling the status quo will involve some amount of additional work for the GP which may further add to their negative emotions. Rather than targeting the GP’s negative emotions it would be more professionally appropriate to target the source of those emotions i.e. rather than targeting GPs’ fear of medico legal consequences, target reducing the risk of medico legal consequences. |
|  | Conserve mental resources | To advise GP on ways of minimising demands on mental resources to facilitate medication review. This behavioural change technique could be applied by encouraging GPs to use guidelines to help them remember the role for certain drugs. However, in multimorbidity, mental resources are required to compute the possible interactions between drugs and diseases, and what potential changes are of value. As no one guideline is available for the myriad combinations of diseases in multimorbidity, facilitating use of mental resources, rather than conservation of mental resources is required. |
|  | Generalisation of a target behaviour | Advise GP to use their approach to medication review in non-multimorbid patients, in a situation involving multimorbidity. Given the particular difficulties relating to polypharmacy, drug-drug and drug-disease interactions reported by GPs in multimorbid patients, the solution will require more than extrapolation of prescribing skills from similar cases to multimorbid ones. |
|  | Action planning (implementation strategy) | Prompt detailed planning of the medication review (must include at least one of time of week, number done together, time of day, with or without patient presentation, triggers for). This is important to give GPs some control over how the intervention is implemented in their practice. As the flexibility of implementation should be seen as an asset in our intervention, this behavioural change technique should be incorporated as an active component. |
|  | Problem solving | Analyse, or prompt the GP to analyse factors influencing their ability to conduct medication reviews and generate or select strategies that include overcoming barriers and/or increasing facilitators. While GPs trying to conduct more medication reviews will have to tailor their approach for their own practice, they are unlikely to have the time or interest in formulating and developing the change strategy themselves. It may work better to develop an intervention and then ask GPs to tailor it for their practice, which is more implementation strategy than problem solving. |
|  | Pros and cons | Advise the person to identify and compare reasons for wanting and not wanting to change their behaviour regarding medication reviews. The qualitative study has already identified that GPs already respect the need to do medication reviews (pros). It has also showed some of the down sides (cons) to medication reviews in patients with multimorbidity/ polypharmacy which lead them to maintaining the status quo instead. The need here is to facilitate medication reviews, rather than just highlight its importance. |
|  | Valued self-identity | Advise the GP to write or complete grading scales about a cherished value or personal strength as a means of affirming their identity as part of a BCS. It is important to empower GPs and improve their sense of self-esteem as professionals in the management of chronic disease, and that may be a useful side effect of any intervention that we undertake. However using this as an active component of the intervention may be perceived by GPs as condescending and viewed with disdain. |
|  | Graded tasks | Set the GP easy to perform tasks, making them increasingly difficult, but achievable until medication review is performed. This may be useful in an educational setting but in routine practice it is not appropriate to stagger the tasks required in medication review: it is important that all medications are reviewed in the context of each other, and the greater bio psychosocial context of the patient. |
|  | Focus on past success | Advise GPs to think about situations in which they previously conducted successful medication reviews. In many cases, these successes may have occurred in an ad hoc fashion, therefore emphasizing their success detracts from the need for systematic, planned medication reviews that we are trying to encourage. This approach may be useful once the medication reviews are underway, to consolidate on going behavioural change. |
|  | Goal setting: behaviour | Set or agree on a goal defined in terms of the conduct of medication review to be achieved. Unsure how much this will achieve, as intention is already there, but competing demands and opportunity cost too great. The greatest part of the behavioural change technique will be to make the target behaviour easier to conduct, rather than simply increasing GPs resolve to do it. |
|  | Goal setting: outcome | Set or agree on a goal defined in terms of a positive outcome of the conduct of medication review – in many patients, there may be no change required to medications, or not potentially inappropriate medications spotted. If focus is on outcomes, and no specific outcomes apparent, this could de-motivate GPs to continue doing medication review. The focus should instead be on the practice of doing medication reviews, regardless of whether inappropriate prescribing is picked up or not. |
|  | Commitment | Ask the GP to affirm or reaffirm statements indicating commitment to change behaviour ( to conduct medication reviews) – although GPs may affirm this, they face many competing demands for their time, so alone, this behavioural change technique will not be effective, and may in fact cause a sense of failure if they do not enact their commitment. |
|  | Self-monitoring | Establish a method for the GP to monitor and record their medication review as part of a behaviour change strategy- as there is no personal gain here, this alone is unlikely to strongly motivate GPs. If it was coupled with some incentive, especially financial incentives, it may be useful. |
|  | Review behavioural goals | GP to review goals for medication review jointly with the person and consider modifying goals or behavioural change strategy in light of current achievement of these goals. This may lead to re-setting the same goal, a small change in that goal or setting a new goal instead of (or in addition to) the first, or no change. Most GPs will not have set own goals for medication review prior to this intervention. |
|  | Review outcome goals | Review the outcome of medication reviews to date jointly with another person and consider modifying goals in light of current achievement. This may lead to changes in GP’s goals regarding the conduct of medication reviews. This may be useful behavioural change techniques once the medication reviews are underway, but there is a high chance that most GPs will not have any medication review goals to review currently. |
|  | Comparative imaginings of future outcomes | Prompt or advise the imagining and comparing of future outcomes of changed (regular or systematic reviews of medications) versus unchanged behaviour (non-systematic reviews of medications. Using data from the qualitative study, future outcomes here include the long-term time-saving nature of regular medication reviews, the lessening of patients risk of adverse effects and less medico-legal risk. While these points would highlight the benefits of doing medication reviews, the imaginings would not be sustainable, and given the competing priorities for GPs in practice, would be unlikely to produce behavioural change. |
| Environmental re-structuring | Prompts/cue | Introduce a stimulus with the purpose of cueing medication review, which would be used at the time of performance of medication review, such as a checklist of things to consider. Could be written or computerised. |
|  | Adding objects to the environment | Add objects to the General Practice environment in order to facilitate performance of medication reviews, involving more than verbal, visual, or written information. The use of Information Technology and Computer Assisted Decision Support Systems is relevant here, and is being researched as an intervention by other groups. |
|  | Restructuring the social environment | Change, or advise to change the social environment in order to facilitate performance of the medication review or create barriers to the unwanted behaviour –failure to conduct medication review. If medication review was scheduled in to regular routine for GPs, and an acceptable activity within the practice for GP to spend dedicated time on, could potentially impact on number of medication reviews conducted in major way – as indicated by qualitative study. |
|  | Restructuring the physical environment | Change, or advise to change the physical environment in order to facilitate performance of medication review or create barriers to the unwanted behaviour (lack of medication review) - other than prompts/cues, rewards and punishments. May not be acceptable to alter GPs working environment physically, and as medication review is a cognitive task, not likely to yield great benefit. |
|  | Associative learning | Present a neutral stimulus jointly with a stimulus that already elicits the behaviour repeatedly until the neutral stimulus elicits that behaviour. No stimulus to prompt medication review already exists, so could not operationalize this. |

1. Michie S, Atkins L, West R: *The Behaviour Change Wheel: A Guide to Desiging Interventions.* Great Britain: Silverback Publishing; 2014.
