# Supplementary material for: Improving medication management in multimorbidity: development of the MultimorbiditY COllaborative Medication Review And DEcision Making (MY COMRADE) intervention using the Behaviour Change Wheel
Source: Implement Sci. 2015 Sep 24;10:132. doi: 10.1186/s13012-015-0322-1 (PMC4582886; doi:10.1186/s13012-015-0322-1)
Supplement: Additional file 3: — BCW step 8: identify mode of delivery using expert panel. (DOCX 33 kb) [file 13012_2015_322_MOESM3_ESM.docx]

# Additional file 3.

# BCW Step 8: Identify mode of delivery using expert panel

The expert panel critically reviewed the format of the emerging intervention strategy, the behavioural change techniques chosen, and the implementation plan. The panel focused on the following aspects of the intervention:

## How should GPs choose patients to initially apply the intervention to?

As GPs cannot be expected to review all patients’ using the format set out in our intervention, they should be informed which type of patients to choose initially. The expert panel considered the following options for patient selection:

- Patient age (i.e. >65years)
- Number of prescribed medications ( i.e. >5 or >10)
- Number of co-morbidities (i.e. >3, >4, etc.)
- Level of patient disability or functional impairment, including care home residence
- Use of high risk medications such as warfarin, non-steroidal anti-inflammatories, diuretics etc.
- GP choice as indicated by GPs discomfort with current medication regimen

With specific reference to the Kings Fund report [[1](#_ENREF_1)] co-authored by two of the expert panel (MD &RP), the panel agreed that GPs be advised to choose patients that were prescribed

- 10 or more regular medicines

OR

- Four or more medicines with:
  - at least one that meets criteria for potentially inappropriate prescribing
  - at risk of a well-recognised potential drug-drug interaction or clinical contraindication
  - difficulties with medicine-taking/ adherence
  - no or only one major diagnosis recorded in the clinical record
  - are receiving end-of-life or palliative care

## What list of prompts should be used to guide the collaborative medication review?

Many instruments are available to assess prescribing.[[2](#_ENREF_2)] The expert panel explicitly considered the following:

- RCGP Prescribing Indicators[[3](#_ENREF_3)]
- Medication Appropriateness Index[[4](#_ENREF_4)]
- Welsh Medicines Support Centre questions[[5](#_ENREF_5)]
- Use of medicine framework: Australia tool [[6](#_ENREF_6)]
- Polypharmacy Guidance, NHS Scotland [[7](#_ENREF_7)]
- STOPP START[[8](#_ENREF_8) ]
- NO TEARS[[9](#_ENREF_9)]

The lack of support felt by GPs in the application of guidelines in multimorbidity was clear in both the systematic review and the qualitative study. In the intervention, the objective of the tool is to prompt the discussion rather than provoke prolonged pharmacological assessment. To this end, it was felt that a broad, generic, pragmatic checklist was most appropriate. Such approaches have been found to improve quality of care in other fields of medicine.[[10](#_ENREF_10)] The last option, NO TEARS[[9](#_ENREF_9)], was originally designed as a generic checklist to underpin *doctor-patient* communication about medications. The seven letters in the acronym prompt the doctor to: review **N**eed and indication for the medication, ask **O**pen questions to the patient on their views on the medication, ensure appropriate **T**ests and monitoring have been conducted, ensure no changes have occured in current **E**vidence and guidelines, ensure patient not experiencing **A**dverse effects, ensure drugs are optimized for **R**isk reduction or prevention, consider **S**implification of medication to improve adherence. As we wanted a set of prompts that would apply to all combinations of diseases seen in multimorbidity we felt the short NOTEARS list, which was not bound by drug or disease, best met our requirements. It would allow consideration of the individual context of each patient case, and give consideration to psychosocial issue that have been shown to be of a greater burden in multimorbid patients [[11](#_ENREF_11)]. To transform this checklist to make it fit for discussion between two GPs, rather than a GP and the patient, the second prompt was modified. Instead of concerning Open questions to the patient, we used **O** to prompt GPs to review whether a patients need for a medication **O**ngoing.

The need to discuss changes with the patient would not be removed – it would just occur downstream from the activities targeted in this intervention.

## What evaluation should take place?

As the goal of the behavioural change intervention was changing GPs behaviour from “maintaining the status quo” to actively reviewing medications, the expert panel agreed that the primary outcome for the initial evaluation would be whether medication reviews were performed using this approach. Each pre-defined BCT included in the intervention would be examined for its role in affecting the desired behaviour. The evaluation questions will be based on the theoretical domains framework: a set of domains that each contains multiple theoretical constructs relating to theory of behavioural change, particularly in the context of healthcare professionals behaviour [[12](#_ENREF_12)]. The evaluation will thus give information on the implementation process, reasons for why the intervention succeeds, fails, or has unexpected consequences, and will identify other causal and contextual mechanisms associated with achieving behavioural change.

As per the MRC, a single primary outcome may not make the best use of the data: a range of measures will be needed and unintended consequences picked up where possible.

Thus, secondary outcomes that will be evaluated in the future were agreed and include:

- Medication related
  - the number of changes recommended in each collaborative medication review
  - the number of changes that are subsequently made to the patients medications
  - the medication appropriateness scores/ number of potentially inappropriate medications before and after the collaborative medication review
- Process of care related
  - number of consultations that directly result from the review
  - the amount of time taken per review
  - the additional workload generated by review – i.e. investigations, referrals etc.

## How should the BCT of action planning be operationalized?

One of the chosen BCTs was action planning which includes implementation planning. This was an important BCT to incorporate into the intervention given the heterogeneous nature of general practice and the wide variations in structures and systems between practices. To allow for these differences, tailoring of the intervention would be required. The expert panel concluded that while the best people to tailor the intervention would be the GPs themselves they must be given clear guidance on how to do this. Thus in order to address barriers to implementation up front, each practice will be asked to consider the following prior to adopting the intervention:

1. What will make this intervention difficult?
2. How should these difficulties be tackled, *knowing your practice*?
3. What is your plan for rolling out this intervention?

- What day? What time of day? Which office? How many at one session? Which GP will you involve? Anything else, specific to how your practice runs?

This process will enhance each participating GPs engagement with the intervention, give them autonomy over how it is rolled out, and highlight potential stumbling blocks before they occur. The practice specific implementation plans will be noted in the evaluation process.

1. Duerden M, Avery A, Payne R: **Polypharmacy and medicines optimisation. Making it safe and sound.** The Kings Fund. London, UK: 2013.

2. Spinewine A, Schmader KE, Barber N, Hughes C, Lapane KL, Swine C, Hanlon JT: **Appropriate prescribing in elderly people: how well can it be measured and optimised?** *Lancet* 2007, **370:**173-184.

3. Spencer R, Bell B, Avery AJ, Gookey G, Campbell SM: **Identification of an updated set of prescribing-safety indicators for GPs.** *British Journal of General Practice* 2014, **64:**e181-e190.

4. Hanlon JT, Schmader KE, Samsa GP, Weinberger M, Uttech KM, Lewis IK, Cohen HJ, Feussner JR: **A method for assessing drug therapy appropriateness.** *J Clin Epidemiol* 1992, **45:**1045-1051.

5. WeMeRecBulletin: **Prescribing for Older People.** In *Welsh Medicines Resource Centre Bulletin*. pp. 4; 2011:4.

6. Scott IA, Gray LC, Martin JH, Mitchell CA: **Minimizing inappropriate medications in older populations: a 10-step conceptual framework.** *Am J Med* 2012, **125:**529-537 e524.

7. NHSScotland: **PolypharmacyGuidance.** In *The Model of Care PolypharmacyWorking Group*.

8. Gallagher P, Ryan C, Byrne S, Kennedy J, O'Mahony D: **STOPP (Screening Tool of Older Person's Prescriptions) and START (Screening Tool to Alert doctors to Right Treatment). Consensus validation.** *International Journal of Clinical Pharmacology and Therapeutics* 2008, **46:**72-83.

9. Lewis T: **Using the NO TEARS tool for medication review.** *BMJ* 2004, **329:**434.

10. Gawande A: *The checklist manifesto : how to get things right.* New York: Metropolitan Books; 2010.

11. Sinnott C, Mc Hugh S, Fitzgerald AP, Bradley C, Kearney PM: **Psychosocial complexity in multimorbidity: the legacy of adverse childhood experiences.** *Fam Pract* 2015, **In press**.

12. Cane J, O'Connor D, Michie S: **Validation of the theoretical domains framework for use in behaviour change and implementation research.** *Implement Sci* 2012, **7:**37.
