# Supplementary material for: Improving medication management in multimorbidity: development of the MultimorbiditY COllaborative Medication Review And DEcision Making (MY COMRADE) intervention using the Behaviour Change Wheel
Source: Implement Sci. 2015 Sep 24;10:132. doi: 10.1186/s13012-015-0322-1 (PMC4582886; doi:10.1186/s13012-015-0322-1)
Supplement: Additional file 4: — Validation of the chosen intervention functions and behavioural change techniques using the theoretical domains framework. (DOCX 32 kb) [file 13012_2015_322_MOESM4_ESM.docx]

**Additional file 4.**

**Validation of the chosen intervention functions and behavioural change techniques using the theoretical domains framework (TDF) [**[**1**](#_ENREF_1)**]**

Table 1 shows the TDF domains deemed relevant to our qualitative data.

Table 2 shows the intervention functions related to these TDF domains. Using the TDF domains relevant to our data to guide the choice of intervention function showed that all of the functions in the Behaviour Change Wheel (BCW) would be potentially useful for our intervention. Thus, the intervention functions indicated by the COM-B behavioural analysis (as described in the main manuscript) are the same as those indicated by the TDF behavioural analysis described here.

Table 3 shows the behavioural change techniques (BCTs) that were ultimately included in the intervention. We mapped these techniques to their relevant TDF domains, using the recent paper by Cane et al.[[2](#_ENREF_2)] and the expert consensus linkage of BCTs and the TDF domains described on page 156 of the Guide to Designing Interventions [[1](#_ENREF_1)]. Reassuringly, the TDF domains associated with the chosen behavioural change techniques had been listed amongst the TDF domains relevant to our qualitative data (shown in Table 1).

**Table1. Determination of relevant intervention functions using the Theoretical Domains Framework**

| COM-B component | Behavioural description and interview source | Potentially relevant TDF domains (constructs) |
| --- | --- | --- |
| Capability-Psychological | **Pharmacological knowledge, an inadequate evidence base, conflicting practice by others, lack of information relevant to general practice reduces GPs capability to do medication reviews**  In some cases, GPs feel there is no available evidence for what is best in multimorbidity  *gp5 “so can we honestly say that this tablet that she has been on X number of years, that by stopping it that she’ll be any better? No we can’t, can we say that by stopping it that it won’t speed up her death? No we can’t”*  *gp7 “It is very hard to justify getting rid of any of his meds, although polypharmacy is a big problem for him.”*  Existing tools/guidelines are not helpful to GPs when conducting medication reviews, and sometimes make things more difficult:  *gp16 “I've yet to see any really decent guidelines, I don't know if they are that useful to be honest in day-to-day decision-making, we prefer to kind of tailor (management)*  *ourselves do you know”*  *Gp7 “with this guy, the guidelines tend to go out the window, because I think if you try to be too strict, if you try to completely adhere to the guidelines with any of his problems then it is going to, adversely affect his other morbidities.”*  Insufficient knowledge on new drugs  *gp3 “we are getting these pieces of information from the drug companies that are nearly impossible to digest, they don’t seem to have any relevance for what I am doing, I find them very hard to read them”*  Information relevant to general practice required  *gp17 “GP led education is what we will do, none of us have any interest in sitting down to a lecture by a nephrologist and more, you know, you don’t have to tell us they are clever”*  Involvement of hospital prescribers can complicate matters in multimorbidity and confuses GPs further  *gp6- “ our consultant hospital colleagues, they are giving the statins out - the Prosper trial seems to be totally ignored, the evidence from it does not seem to be taken on board.”*  *gp6 “(patients)are never strictly in the right boxes; there are always the complicating factors; there’s always the, you know, diabetes with the gout - and you send them up to a rheumatologist they come back with a huge dose of steroid then, you say ‘well I could have done that’”*  *gp10“when Dr XX put her on a big whack of steroids, this women is a diabetic, and there was no reference to the fact that she was diabetic - the adjustments that would need to be made, you know?”* | Knowledge (knowledge)  Skills (skills, practice, competence)  Memory/ attention/ decision making processes (attention, decision making, cognitive overload) |
| Opportunity- Physical | **GPs feel they do not have adequate time resources to conduct medication reviews**  Lack of time for renewing scripts within the consultation.  *gp13 “there are times when you kind of have to say to someone when they come in ‘I’ll have to do some of this another day, or you’ll have to come back to me, we’ll do it in a different structure in a different format”*  *gp1 “if I just had time to have a 30 -45 minute consultation with a patient while you don’t have the waiting room building up, you could actually get to the bottom of some of the stuff they’re on”*  Lack of systems within the practice that allocate time to the activity of medication review  *gp9 “it’s one of the old chestnuts is that you are so busy when you are working that to take the time to look at these things in proper, I mean if you are going to do it, you have to do it obviously properly”*  *gp12 “sometimes it would be nice to start afresh and I could ask the patient to come into me instead of them coming to me with some big long thing or whatever they had wrong with them today; instead me saying to them ‘now this is what I want to talk about (sorting out medications)’”* | Environmental context and resources (organisational culture, resources/material resources, barriers and facilitators)  Goals (action planning) |
| Opportunity- Social | **GPs feel that conducting medication reviews is complicated by the lack of social convention or acceptability, from a patient’s perspective, of having medications removed or rationalized.**  Patient attachment to medications  *gp11 “She’s attached to them, so I haven’t, I haven’t had the heart to broach it”*  *gp7 “She wasn’t keen to change her Risperdal because she had been on it for years”*  *gp5- “ they say ‘oh god, doc, I want to stay on that’ even if you feel it’s doing feck all good you’ll just prescribe it out again, you know- who are we to say ‘no, no we need to stop that’ do you know”*  *gp9 “she would be the type of patient, I would think, where you’d maybe get a phone call the following week saying ‘I don’t feel as well off that tablet as I did on it’ in a lot of cases you’d end up putting her back on it.”*  Patients’ misconceptions about longstanding medications  *gp13 – “some of the stuff she is on like the domperidone and the betahistine and stuff I’m not really convinced that she needs it. I have talked to her about it - about whether or not it might be useful to take things off but she’s reluctant to take them out and as far as she is concerned they’ve been started at some point over the years for her for some reason, so she wants to try and keep them”* | Social influences (social pressures, social norms) |
| Motivation- Automatic | **Reflex responses to polypharmacy in multimorbid patients, who demonstrate no obvious adverse drug effects, was to “maintain the status quo” in almost all interviews. This occurred due to lack of time in the consultation, lack of consistency in hospital prescribers, lack of convention for stopping medications, and lack of confidence in own prescribing.**  gp1 “maintain the status quo’  gp11 “she has been doing better than she has been in a long time so I’m not going to rock the boat at all”  gp12 “‘look she’s on it, she’s fine, it doesn’t bother her, it’s suiting her fine”  gp13 “largely for her I’d let it sit, I think if she is stable I don’t try and change too much”  gp14 “there is that aspect of not rocking the boat, you know and being straight up about it as well, sometimes as well you can get into the routine ‘oh are you just in for the prescription?’, you just print it off automatically without giving due consideration to can we shorten this, can we do this that and the other.”  gp18 “take the line of least resistance! Here’s another 3 months prescription for it!!”  gp19 “anything that complex I really didn’t entertain changing because why stir up?” | Reinforcement (rewards, incentives) |
| Motivation- Reflective | **MOTIVATIONS AGAINST MEDICATION REVIEW**  **GPs have beliefs about negative consequences of medication rationalisation such as potential medico-legal repercussions, negative responses from patient/next of kin, and harming the doctor-patient relationship due to risk of patient perception of medication rationalization as withdrawal of care.**  Gp9 “would be loath to stop it, again probably in that situation it’s probably medico-legal, if you stop it and they do get a thrombosis the next week, you will feel a bit guilty”  Gp6 “his wife or he will say ‘hang on a second I want to go on as long as I can, why are you risking me getting a heart attack?’ Why stop my aspirin and my statin, if there is a small risk I’ll get a heart attack, why not leave me on it, why are you taking me off’”  Leaving decisions to other clinicians: gp16 “I'd prefer to have them (specialists) say yes or no, because that way at least if I get sued I've covered myself.”  **Some GPs have negative beliefs about their capabilities relative to other prescribers, and find it difficult to stop what others have started (low self-efficacy / empowerment)**  Gp6 “I find that in some of the situations that the patient comes to you, they’ve been in hospital, something happens they end up in hospital but when they come out, they come out on medications that I would not have necessarily have started”  Gp9 “I suppose it’s deference to consultant opinion as usual, I suppose I should probably read up about it again and see whether I can think of reducing it.” and “the problem is, I suppose, in terms of cardiac stuff and in particular anti-angina stuff you have to be very brave to stop that I think, in a lot of ways.”  gp13 “I’m absolutely in fear of changing these medications at all (shakily laughing) - like she goes into heart failure every so often and I don’t really know where to go when it happens”  **The opportunity cost of medications reviews is using that time for other purposes, some of which are associated with greater gains (financial / time efficiency / delivering patient determined rather than doctor determined care)**  Lack of remuneration for changing medications:  *Gp17 “at some point I have invested as much time as I can, in to them, and don’t forget this is all pro bono, and you know, sometime you say ‘will I keep doing it?”*  *Gp11: “she has had multiple other things going on as well, so the consultation time would be taken up (if medications were also reviewed)”*  *Gp6 “to really get him on the amount of medication he needs, we’d be seeing him almost every few weeks - we’d be seeing him very frequently and that has huge implications because you have so many patients and you can’t, if you saw everybody every few weeks, you can’t do it”*  **MOTIVATIONS TO REVIEW MEDICATIONS**:  **GPs also have beliefs about the consequences of not reviewing medications that could be used to motivate them to do reviews:**  Demonstrating that medications have been reviewed is important medico-legally:  *gp19 “It is your signature on the GMS prescription so if you haven’t weighed up the pros and cons, and made a decision yourself, even though someone else started it, if they end up addicted to such and such a thing, you’re responsible”*  *gp10 “What is important in theory and what is actually important in practice, on the ground, are often two entirely different things; but medico-legally the problem is that if this guy dies of renal failure they are going to be looking at his medication list and you will be thinking ‘oh, crap’”*  It is good defensible practice to do and document medication reviews:  *gp10 “the longer I am in practice the longer my clinical notes are getting and the more I am documenting; aware of interaction, need to watch renal function but that must balance benefits versus risks.”*  Important to review medications in order to discuss implications of polypharmacy with patients:  *gp10“Everything interacts with everything these days and you explain to them ‘look, technically you are not supposed to be on that but look it’s working for you’”*  Negative emotions about not reviewing medications, could be alleviated by reviewing them:  *gp11 “it would make you feel nervous, because obviously you wouldn’t like anything happening somebody, and she probably was on it too long, it would have been difficult for me to probably stand over it... I could have probably been in trouble myself if something had happened her”*  *gp8 “He was on something else, I think it was a PPI and it was interfering with his HIV and I felt very bad about that after, because when he came out of hospital, i thought, oh my god”*  Increasing comfort with prescribing if reviewed systematically:  *gp17 “we try and do a three month chart review on diabetics to make sure that we have pulled all of them in and they are as up to date as we can get them, so the plan is that everybody has all the boxes ticked, so now I’m quite comfortable with diabetes, I’m quite comfortable with hypertension”*  The desire to practice best medicine, avoid medication errors and reduce patients treatment burden. | Social/ professional role and identity (Professional role & identity)  Beliefs about capabilities (self-efficacy, empowerment, professional confidence)  Beliefs about consequences (anticipated regret or consequences) |

**Table 2. Mapping the relevant TDF domains to their related intervention function [**[**1**](#_ENREF_1)**]**

| Relevant TDF domains (with relevant constructs) | Potentially relevant intervention function |
| --- | --- |
| Knowledge (knowledge) | Education |
| Skills (skills, practice, competence) | Training |
| Memory/ attention/ decision making processes (attention, decision making, cognitive overload) | Training  Environmental Restructuring  Enablement |
| Environmental context and resources (organisational culture, resources/material resources, barriers and facilitators) | Training  Restriction  Environmental Restructuring  Enablement |
| Goals (action planning) | Education  Persuasion  Incentivisation  Coercion  Modelling  Enablement |
| Social influences (social pressures, social norms) | Restriction  Environmental Restructuring  Modelling  Enablement |
| Reinforcement (rewards, incentives) | Training  Incentivisation  Coercion  Environmental Restructuring |
| Social/ professional role and identity (Professional role & identity) | Education  Persuasion  Modelling |
| Beliefs about capabilities (self-efficacy, empowerment, professional confidence) | Education  Persuasion  Modelling  Enablement |
| Beliefs about consequences (anticipated regret or consequences) | Education  Persuasion  Modelling |

**Table 3. Mapping the behavioural change techniques eventually chosen for the intervention back to the TDF domains they relate to, using the Guide to Designing Interventions[**[**1**](#_ENREF_1)**] and the paper by Cane et al[**[**2**](#_ENREF_2)**]**

| Chosen intervention function | Chosen behaviour change technique | Related TDF domains [[2](#_ENREF_2)] |
| --- | --- | --- |
| Environmental Restructuring | Restructuring the social environment✓  Prompts/cue✓ | Environmental context & resources  Environmental context & resources |
| Enablement | Social support (practical)-✓  Action planning (implementation intentions)✓ | Social influences  Goals |
| Incentivisation | Self-reward or incentive – CME✓ | Reinforcement |

1. Michie S, Atkins L, West R. The Behaviour Change Wheel: A Guide to Desiging Interventions. Great Britain: Silverback Publishing; 2014.

2. Cane J, Richardson M, Johnston M, Ladha R, Michie S. From lists of behaviour change techniques (BCTs) to structured hierarchies: Comparison of two methods of developing a hierarchy of BCTs. Br J Health Psychol. 2015;20:130-50.
